# Candida albicans Double Mutants Lacking both *EFG1* and *WOR1* Can Still Switch to Opaque

**DOI:** 10.1128/mSphere.00918-20

**Published:** 2020-09-23

**Authors:** Yang-Nim Park, Claude Pujol, Deborah J. Wessels, David R. Soll

**Affiliations:** a Developmental Studies Hybridoma Bank, Department of Biology, The University of Iowa, Iowa City, Iowa, USA; University of Georgia

**Keywords:** *Candida albicans*, *Candida albicans* mating, *EFG1*, *WOR1*, gray phenotype, opaque phenotype switching

## Abstract

Candida albicans, a pervasive opportunistic pathogen, undergoes a unique phenotypic transition from a “white” phenotype to an “opaque” phenotype. The switch to opaque impacts gene expression, cell morphology, wall structure, metabolism, biofilm formation, mating, virulence, and colonization of the skin and gastrointestinal (GI) tract. Although the regulation of switching is complex, a paradigm has evolved from a number of studies, in which, in its simplest form, the transcription factors Efg1 and Wor1 play central roles. When *EFG1* is upregulated under physiological conditions, it represses *WOR1*, an activator of white-to-opaque switching, and the cell expresses the white phenotype; when *EFG1* is downregulated, *WOR1* is derepressed and activates expression of the opaque phenotype. Deletion of either *EFG1* or *WOR1* supports this yin-yang model of regulation. Here, we demonstrate that this simple model is insufficient, since strains in which *WOR1* and *EFG1* are simultaneously deleted can still be induced to switch *en masse* from white to opaque. Opaque cells of double mutants (*efg1^−/−^ wor1^−/−^*) are enlarged and elongate, form an enlarged vacuole, upregulate *mCherry* under the control of an opaque-specific promoter, form opaque cell wall pimples, express the opaque phenotype in lower GI colonization, and, if *MTL* homozygous, form conjugation tubes in response to pheromone and mate. These results can be explained if the basic and simplified model is expanded to include a *WOR1*-independent alternative opaque pathway repressed by *EFG1*.

**IMPORTANCE** The switch from white to opaque in Candida albicans was discovered 33 years ago, but it is still unclear how it is regulated. A regulatory paradigm has emerged in which two transacting factors, Efg1 and Wor1, play central roles, Efg1 as a repressor of *WOR1*, which encodes an activator of the transition to the opaque phenotype. However, we show here that if both *EFG1* and *WOR1* are deleted simultaneously, bona fide opaque cells can still be induced *en masse*. These results are not compatible with the simple paradigm, suggesting that an alternative opaque pathway (AOP) exists, which can activate expression of opaque and, like *WOR1*, is repressed by *EFG1*.

## Abstract (precis)

The switch from white to opaque in Candida albicans was discovered 33 years ago, but it is still unclear how it is regulated. A regulatory paradigm has emerged in which two transacting factors, Efg1 and Wor1, play central roles, Efg1 as a repressor of *WOR1*, which encodes an activator of the transition to the opaque phenotype. However, we show here that if both *EFG1* and *WOR1* are deleted simultaneously, bona fide opaque cells can still be induced *en masse*. These results are not compatible with the simple paradigm, suggesting that an alternative opaque pathway (AOP) exists, which can activate expression of opaque and, like *WOR1*, is repressed by *EFG1*.

## INTRODUCTION

Candida albicans, a highly successful opportunistic yeast pathogen, undergoes a reversible transition between a “white” and “opaque” phenotype that impacts both colony and cellular morphology in a profound fashion (1, 2). In 2002, Miller and Johnson discovered that in order to switch, C. albicans strains, which in nature are >95% **a**/α, undergo homozygosis at the *MTL* locus (3, 4). In the same study, Miller and Johnson also discovered that in order to mate, *MTL*-homozygous strains had to switch from white to opaque (3, 4). Sonneborn et al. (5) then demonstrated that the transcription factor (TF) Efg1 was a repressor of switching. Three laboratories then simultaneously reported that the TF Wor1 was an activator of switching (6–8). Based primarily on chromatin immunoprecipitation with microarray technology (ChIP-chip) to identify promoter binding sites in *MTL*-homozygous strains, models of regulatory networks of multiple interacting TFs were then developed for the differential regulation of *EFG1* and *WOR1* (9–13). The assumption that white-opaque switching was restricted to *MTL*-homozygous strains persisted until 2013, when it was reported (14) that approximately one-third of a collection of clinical **a**/α isolates could be induced to switch. This result was reinforced by additional studies of clinical **a**/α isolates (15). The **a**/α opaque cells, however, differed from **a/a** and α/α opaque cells in that they were mating incompetent (14).

To date, the simple “yin-yang” perception of the regulation of white-opaque switching by Efg1 and Wor1 has persisted. Deletion not only of *WOR1* alone (11, 16, 17) but also of both *WOR1* and *EFG1* in double mutants (11, 16, 17) was reported to block the transition from white to opaque. However, in an earlier report by Nie et al. (18), evidence was presented that at 22°C in air, with glucose as the carbon source, at pH 6.8, double deletion mutants of *EFG1* and *WOR1* (*efg1*Δ/Δ *wor1*Δ/Δ) formed cells that were opaque like. However, the conditions employed in this early study (18) were then shown in later studies to favor the formation of a “gray” rather than opaque cell (15, 19). Gray cells expressed *OP4*, but at greatly diminished levels, did not form the unique opaque cell wall pimples, and exhibited a tiny elongate or an intermediate phenotype, the latter elongate but of similar size to that of the white (15, 16, 19, 20). These observations raised the possibility that Nie et al. (18) may have identified the intermediate gray phenotype, rather than the opaque phenotype, in *efg1*Δ/Δ *wor1*Δ/Δ double mutants. Moreover, Nie et al. (18) described elongate cells that were the same size as white cells, not enlarged like opaque cells, and had levels of *OP4* expression consistent with the reduced levels of expression described by Tao et al. (16) for gray cells. As we show here by repeating the protocols of Nie et al. (18), expression of *GFP*, regulated by the promoter of the gray cell-specific gene *HSP31*, is upregulated in the cell preparation, *mCherry*, regulated by the promoter of the opaque cell-specific gene *OP4*, is downregulated, and the phenotype is that of the intermediate gray cell.

Therefore, we revisited the question of whether *efg1*Δ/Δ *wor1*Δ/Δ double mutants, generated in both *MTL*-heterozygous and *MTL*-homozygous strains, could switch to opaque. The results we present demonstrate that *efg1*Δ/Δ *wor1*Δ/Δ double mutants of the **a**/α strains SC5314 and P37039, an **a**/− derivative of SC5314, a −/α derivative of SC5314, and two **a/a** clinical strains can be induced to switch *en masse* to a bona fide opaque phenotype. In addition, opaque cells of *MTL*-hemizygous or *MTL*-homozygous double mutants, but not *MTL*-heterozygous double mutants, formed conjugation tubes in response to pheromone of the opposite mating type and underwent mating. Finally, *efg1*Δ/Δ *wor1*Δ/Δ double mutants of **a**/α, −/α, and **a/a** strains outcompeted parental wild-type strains in a mouse model for gastrointestinal colonization and expressed the opaque phenotype at the site of colonization, as previously demonstrated for *efg1*Δ/Δ *WOR1^+/+^* mutants (15). The results suggest the existence of a redundant “alternative opaque pathway” (AOP), repressed by Efg1, which is derepressed in the double mutant *efg1^−/−^ wor1^−/−^*.

## RESULTS

### Double deletion mutants *efg1*Δ/Δ *wor1*Δ/Δ in a/α strains.

**FIG 1:**
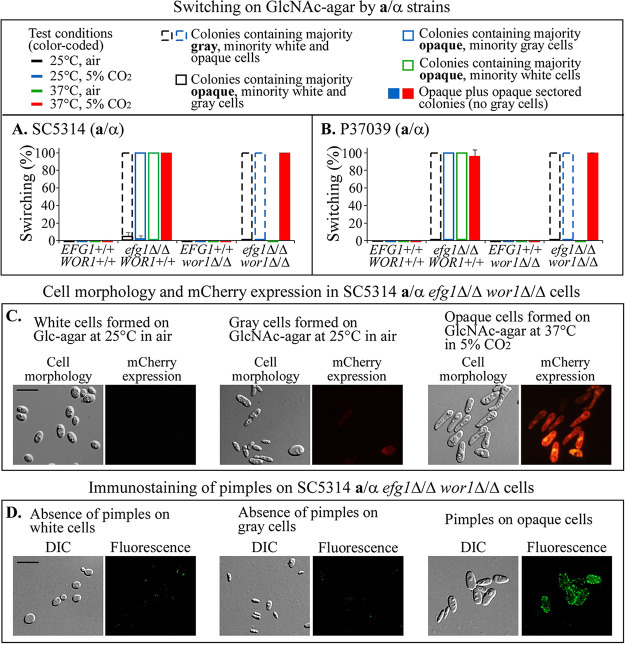
Switching by wild type and single and double deletion mutants of *EFG1* and *WOR1* in two clinical **a**/α strains. Switching from white to opaque and gray was assessed on GlcNAc agar under four tested sets of environmental conditions (top left). (A) Switching frequencies of strain SC5314 **a**/α wild type and deletion mutants. (B) Switching frequencies of strain P37039 **a**/α wild type and deletion mutants. On average, >500 colonies were assessed in each of three experiments. Error bars represent the standard deviations of the means from the three experiments. The key to the switching frequencies graphed in panels A and B is shown at the top. (C) Cell morphology and *mCherry* expression in the SC5314 **a**/α *efg1*Δ/Δ *wor1*Δ/Δ double mutant for white, gray, and opaque cells. *mCherry* expression was under the regulation of the promoter of the opaque-specific gene *OP4*. (D) Immunostaining of opaque cell wall pimples with anti-pimple antiserum in the SC5314 **a**/α *efg1*Δ/Δ *wor1*Δ/Δ double mutant for white, gray, and opaque cells. Scale bars, 10 μm.

The two wild-type (wt) **a**/α parental strains, SC5314 (21) and P37039 (22), which are *EFG1^+/+^ WOR1^+/+^*, did not form either gray or opaque cells when plated on agar containing modified Lee’s medium (23, 24) with 1.25% glucose as the sugar source at pH 6.8 (Glc agar) or on agar containing modified Lee’s medium with 2% *N*-acetylglucosamine as the sugar source at pH 6.8 (GlcNAc agar) under any of the four tested sets of environmental conditions (25°C, air; 25°C, 5% CO_2_; 37°C, air; and 37°C, 5% CO_2_) (15, 19) (Fig. 1A and B). Neither white cells of the two *efg1*Δ/Δ *WOR1^+/+^* and *EFG1^+/+^ wor1*Δ/Δ single mutants nor white cells of the *efg1*Δ/Δ *wor1*Δ/Δ double mutants, derived from the two wt **a**/α strains, switched to gray or opaque on Glc agar under any of the four tested sets of conditions at pH 6.8. Therefore, in the remainder of this report, we will present primarily the results of the studies performed on GlcNAc agar, unless noted otherwise.

On GlcNAc agar, cells of both of the wt parental **a**/α strains (*EFG1^+/+^ WOR1^+/+^*) and their *wor1*Δ/Δ single mutants (*EFG1^+/+^ wor1*Δ/Δ) did not switch to either the gray or opaque phenotype under any of the four sets of environmental conditions (Fig. 1A and B). At 25°C in air, both **a**/α *efg1*Δ/Δ *WOR1^+/+^* mutant strains formed colonies containing a majority of gray cells, a minority of white cells, and a small minority of opaque cells (Fig. 1A and B) (15, 19). At 25°C in 5% CO_2_, the **a**/α *efg1*Δ/Δ *WOR1^+/+^* cells formed colonies containing mainly opaque cells with a minority containing gray and white cells, and a very small minority of opaque colonies contained exclusively opaque cells (Fig. 1A and B) (15, 19). At 37°C in air, **a**/α *efg1*Δ/Δ *WOR1^+/+^* cells formed colonies containing a majority of opaque cells and a minority of white cells (Fig. 1A and B) (15, 19). Finally, at 37°C in 5% CO_2_, the majority of colonies formed by **a**/α *efg1*Δ/Δ *WOR1^+/+^* cells were homogenous opaque colonies containing exclusively opaque cells, and a minority of white colonies had opaque sectors, the sectors containing exclusively opaque cells (Fig. 1A and B). It should be pointed out first, and as previously noted (15, 19), that *efg1*Δ/Δ *WOR1^+/+^* cells of both **a**/α strains formed colonies containing gray cells at 25°C but not at 37°C. Second, both *efg1*Δ/Δ *WOR1^+/+^* mutants formed opaque cells at both 25°C and 37°C (Fig. 1A and B).

On GlcNAc agar at 25°C in air, 100% of the colonies formed by both *efg1*Δ/Δ *wor1*Δ/Δ double mutants contained a majority of gray cells and a minority of white and opaque cells, very similar to that by the single *efg1*Δ/Δ mutants (Fig. 1A and B). At 25°C in 5% CO_2_, the colonies formed by both double mutants again contained a majority of gray cells and a minority of white and opaque cells (Fig. 1A and B), which differed from colonies formed by the *efg1*Δ/Δ *WOR1^+/+^* mutants, which formed colonies containing a majority of opaque cells (Fig. 1A and B). Surprisingly, at 37°C in air, the **a**/α *efg1*Δ/Δ *wor1*Δ/Δ double mutants formed exclusively white colonies containing white cells, in contrast to colonies formed by *efg1*Δ/Δ *WOR1^+/+^* mutants, which contained a majority of opaque cells and a minority of white cells (Fig. 1A and B). Finally, on GlcNAc agar at 37°C in 5% CO_2_, 100% of colonies formed by the double mutants were opaque and a minority were sectored, and the opaque colonies and opaque sectors contained exclusively opaque cells, the same response as the single *efg1*Δ/Δ *WOR1^+/+^* mutant derivatives (Fig. 1A and B). The switching data for **a**/α wild type and derivative deletion mutants in Fig. 1A and B are synopsized in Table 1.

**TABLE 1:** Synopsis of the results of mutant analysis under the four sets of environmental conditions^*a*^

Strain	Genotype	GlcNAc agar			
		25°C		37°C	
		Air	CO_2_	Air	CO_2_
**a**/α					
SC5314	*EFG1^+/+^ WOR1^+/+^*	Wh	Wh	Wh	Wh
	*efg1*Δ/Δ *WOR1^+/+^*	Gr (wh, op)	Op (wh, gr)	Op (wh)	Op
	*EFG1^+/+^ wor1*Δ/Δ	Wh	Wh	Wh	Wh
	*efg1*Δ/Δ *wor1*Δ/Δ	Gr (wh, op)	Gr (wh, op)	Wh	Op
P37039	*EFG1^+/+^ WOR1^+/+^*	Wh	Wh	Wh	Wh
	*efg1*Δ/Δ *WOR1^+/+^*	Gr (wh, op)	Op (wh, gr)	Op (wh)	Op
	*EFG1^+/+^ wor1*Δ/Δ	Wh	Wh	Wh	Wh
	*efg1*Δ/Δ *wor1*Δ/Δ	Gr (wh, op)	Gr (wh, op)	Wh	Op
**a**/−					
SC5314	*EFG1^+/+^ WOR1^+/+^*	Wh	Op	Wh	Op
	*efg1*Δ/Δ *WOR1^+/+^*	Op	Op	Op	Op
	*EFG1^+/+^ wor1*Δ/Δ	Wh	Wh	Wh	Wh
	*efg1*Δ/Δ *wor1*Δ/Δ	Gr (wh, op)	Op	Op	Op
−/α					
SC5314	*EFG1^+/+^ WOR1^+/+^*	Wh	Op	Wh	Op
	*efg1*Δ/Δ *WOR1^+/+^*	Op	Op	Op	Op
	*EFG1^+/+^ wor1*Δ/Δ	Wh	Wh	Wh	Wh
	*efg1*Δ/Δ *wor1*Δ/Δ	Gr (wh, op)	Op	Op	Op
**a**/**a**					
P37005	*EFG1^+/+^ WOR1^+/+^*	Wh	Op	Wh	Op
	*efg1*Δ/Δ *WOR1^+/+^*	Op	Op	Op (wh)	Op
	*EFG1^+/+^ wor1*Δ/Δ	Wh	Wh	Wh	Wh
	*efg1*Δ/Δ *wor1*Δ/Δ	Gr (wh, op)	Op	Op (wh)	Op
P94015	*EFG1^+/−^ WOR1^+/+^*	Wh	Op	Wh	Op
	*efg1^−/−^ WOR1^+/+^*	Op	Op	Op	Op
	*EFG1^+/−^ wor1*Δ/Δ	Wh	Wh	Wh	Wh
	*efg1^−/−^ wor1*Δ/Δ	Gr (wh, op)	Op	Op	Op

a In all cases, white cells were plated on GlcNAc agar. Major phenotypes: Wh, white; Gr, gray; Op, opaque. Minor phenotypes: wh, white; gr, gray; op, opaque.

### Opaque cell phenotype of a/α *efg1*Δ/Δ *wor1*Δ/Δ double mutants.

To verify that **a**/α *efg1*Δ/Δ *wor1*Δ/Δ cells expressed a bona fide opaque phenotype, we analyzed size and shape (2, 25, 26), presence of a large vacuole, upregulation of *mCherry* under the control of the promoter of opaque-specific gene *OP4* (27), and the formation of opaque-specific cell wall pimples (26), all characteristics of bona fide opaque cells. White cells formed by SC5314 **a**/α *efg1*Δ/Δ *wor1*Δ/Δ cells on Glc agar at 25°C in air were round to ellipsoidal (Fig. 1C), did not express *mCherry* (Fig. 1C), did not contain an enlarged vacuole, and did not form cell wall pimples, which were assessed by immunofluorescent staining with a pimple-specific antibody (26) (Fig. 1D). Gray cells formed by SC5314 **a**/α *efg1*Δ/Δ *wor1*Δ/Δ cells on GlcNAc agar at 25°C in air were either small and elongate (“tiny elongate” phenotype) or intermediate in size and elongate (“transition” phenotype) (Fig. 1C), as previously described (15, 16, 19). The gray cells did not express *mCherry* (Fig. 1C) or form opaque-specific cell wall pimples (Fig. 1D). Opaque cells, formed by *efg1*Δ/Δ *wor1*Δ/Δ cells on GlcNAc agar at 37°C in 5% CO_2_, the set of conditions causing mass conversion to opaque (Fig. 1A and B), were large, elongate, and asymmetric (i.e., one end wider than the other) (Fig. 1C), contained an enlarged vacuole, expressed *mCherry* regulated by an opaque-specific promoter (Fig. 1C), and stained for opaque cell wall pimples (Fig. 1D). These results demonstrate that **a**/α *efg1*Δ/Δ *wor1*Δ/Δ double mutants can be induced to form bona fide opaque cells.

### Double deletion mutants in a/−, −/α, and a/a strains.

To test whether *MTL*-hemizygous (**a**/**−** or **−**/α) and *MTL*-homozygous (**a/a**) *efg1*Δ/Δ *wor1*Δ/Δ double mutants can form opaque cells, we generated single and double deletion mutants of *EFG1* and *WOR1* in four strains (see Table S1 in the supplemental material).

10.1128/mSphere.00918-20.1 TABLE S1 Strains used in this study. Download Table S1, PDF file, 0.4 MB. Copyright © 2020 Park et al. 2020 Park et al. This content is distributed under the terms of the Creative Commons Attribution 4.0 International license.

**FIG 2:**
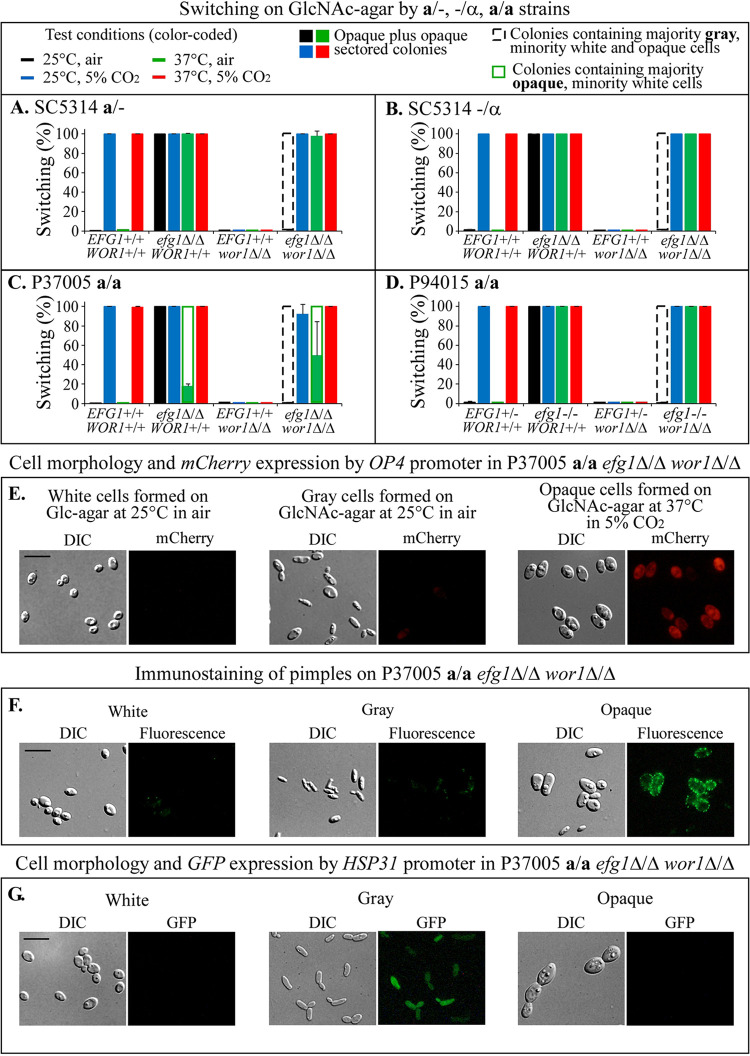
Switching by wild type and single and double deletion mutants of *EFG1* and *WOR1* in *MTL*-hemizygous and *MTL*-homozygous strains. Switching was assessed on GlcNAc agar under four tested sets of environmental conditions (top left). (A) Switching frequencies of SC5314 **a**/− strain and deletion mutants. (B) Switching frequencies of SC5314 −/α strain and deletion mutants. (C) Switching frequencies of P37005 **a/a** strain and deletion mutants. (D) Switching frequencies of the P94015 **a/a** strain *EFG1^+/−^ WOR1^+/+^* (generated from the clinical isolate P94015 **a/a** *efg1^−/−^ WOR1^+/+^* by complementation with a wild type copy of *EFG1*) and single and double mutants of *EFG1* and *WOR1*. On average, >500 colonies were assessed in each of three experiments. Error bars represent the standard deviations of the means from the three experiments. The key to switching frequencies graphed in panels A to D is shown at the top. (E) Cell morphology and *mCherry* expression in P37005 **a**/**a** *efg1*Δ/Δ *wor1*Δ/Δ double mutant for white, gray, and opaque cells. *mCherry* expression was under the regulation of the opaque-specific gene *OP4* promoter. (F) Immunostaining with anti-pimple antiserum of white and opaque cells of the P37005 **a**/**a** *efg1*Δ/Δ *wor1*Δ/Δ double mutant for white, gray, and opaque cells. (G) Cell morphology and *GFP* expression in P37005 **a**/**a** *efg1*Δ/Δ *wor1*Δ/Δ double mutant for white, gray, and opaque cells. *GFP* was under the regulation of the gray-specific gene *HSP31* promoter. Scale bars, 10 μm.

None of the tested *MTL*-hemizygous or *MTL*-homozygous strains harboring wild-type alleles of both *EFG1* and *WOR1* switched significantly (i.e., >2%) on GlcNAc agar to opaque in air at either 25°C or 37°C (Fig. 2A to D). However, they formed opaque or opaque sectored colonies *en masse* (100%) in 5% CO_2_, at either 25°C or 37°C (Fig. 2A to D). *MTL*-hemizygous and -homozygous mutants lacking *WOR1* (*EFG1^+/+^ wor1*Δ/Δ or *EFG1^+/−^ wor1*Δ/Δ) did not switch to either opaque or gray under any of the tested conditions (Fig. 2A to D), which was similar to that for **a**/α *EFG1^+/+^ wor1*Δ/Δ single mutants (Fig. 1A and B). All of the *MTL*-hemizygous or *MTL*-homozygous *efg1*Δ/Δ single mutants (*efg1*Δ/Δ *WOR1^+/+^* and *efg1^−/−^ WOR1^+/+^*) switched to opaque *en masse* under all of the four tested conditions (Fig. 2A to D). However, at 37°C in air, the *efg1*Δ/Δ *WOR1^+/+^* single mutant of strain P37005 exhibited a reduced switching efficiency.

On GlcNAc agar at 25°C in air, all four *MTL*-hemizygous and -homozygous *efg1*Δ/Δ *wor1*Δ/Δ or *efg1^−/−^ wor1*Δ/Δ double mutants formed colonies containing a majority of gray cells and a minority of white and opaque cells (Fig. 2A to D). However, at 25°C in 5% CO_2_, 37°C in air, and 37°C in 5% CO_2_, the double mutants formed opaque colonies *en masse* (Fig. 2A to D). Similar to the switching of the *efg1*Δ/Δ single mutant of P37005 strain at 37°C in air, the double mutant exhibited a reduced switching efficiency at 37°C in air (Fig. 2C). The switching data for *MTL*-hemizygous and *MTL*-homozygous strains in Fig. 2A to D are synopsized in Table 1.

### Opaque cell phenotype of a/−, −/α, and a/a double mutants.

Opaque cells of the *MTL*-hemizygous (**a**/− or −/α) and *MTL*-homozygous (**a/a**) *efg1*Δ/Δ *wor1*Δ/Δ or *efg1^−/−^ wor1*Δ/Δ double mutants exhibited the cellular characteristics of wild type *MTL*-heterozygous and *MTL*-homozygous wild-type opaque-phase cells. The opaque cells were enlarged and elongate (Fig. 2E), expressed *mCherry* under the regulation of the opaque-specific *OP4* promoter (Fig. 2E), and formed opaque-specific cell wall pimples that stained with an opaque-specific antibody (Fig. 2F) but did not express *GFP* under the regulation of the gray-specific *HSP31* promoter (Fig. 2G).

### Repeat of a previous study of a double mutant.

**FIG 3:**
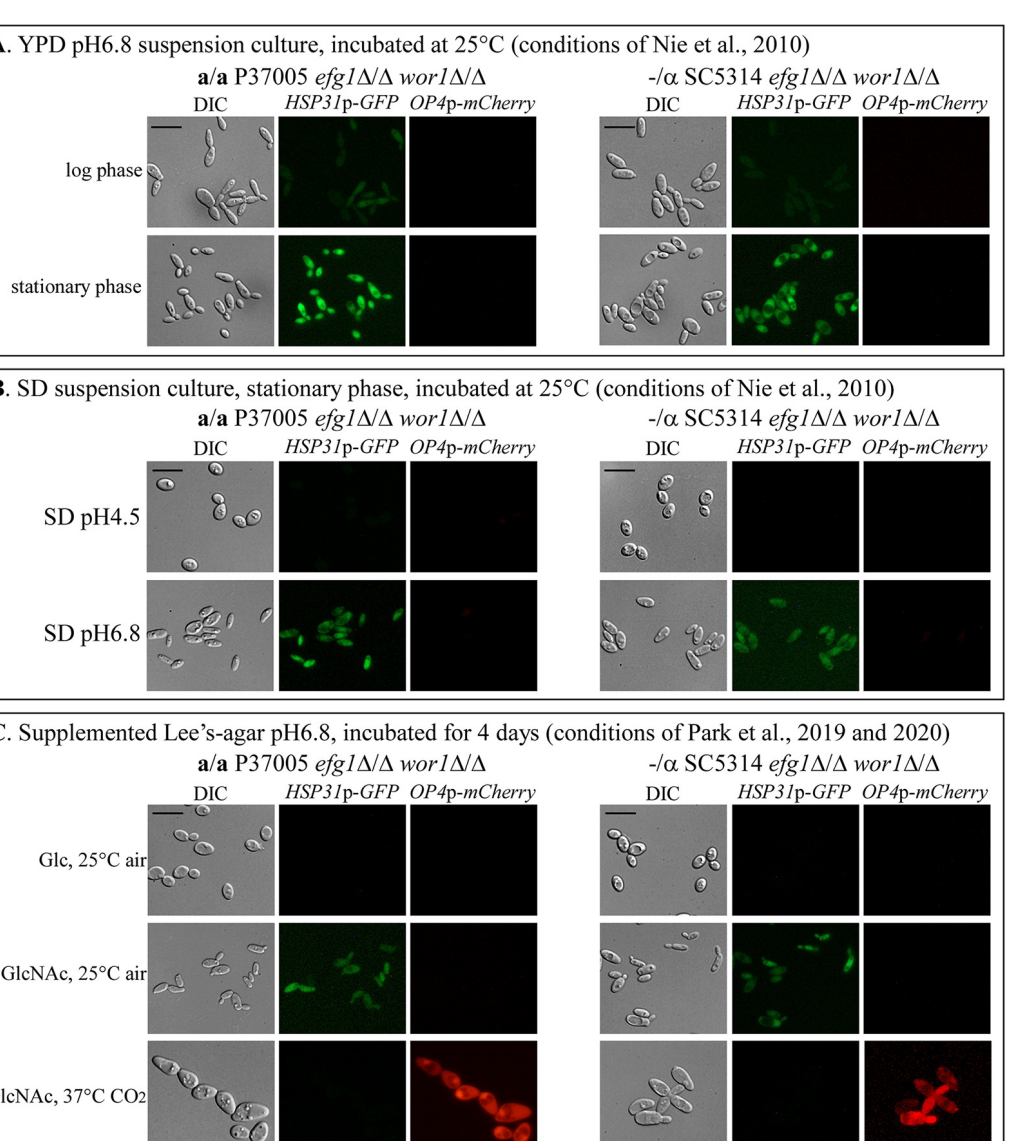
The phenotype of cells of **a**/**a** and −/α *efg1*Δ/Δ *wor1*Δ/Δ double mutants grown in suspension cultures in YPD or SD medium. Nie et al. (18) analyzed double mutants under conditions conducive to gray rather than opaque cell formation. We therefore tested cell phenotype under their conditions and confirmed that the conditions induced the gray, not opaque, cell phenotype. We analyzed two *efg1*Δ/Δ *wor1*Δ/Δ double mutants that harbored *GFP* under the regulation of the gray-specific *HSP31* promoter and *mCherry* under the regulation of the opaque-specific *OP4* promoter. (A) Cell morphology and expression of *GFP* and *mCherry* for cells from log- and stationary-phase suspension cultures grown in YPD (pH 6.8) medium at 25°C in air. (B) Cell morphology and expression of *GFP* and *mCherry* for cells were from stationary-phase suspension cultures grown in SD (pH 6.8 or pH 4.5) medium at 25°C in air. (C) Cell morphology and expression of *GFP* and *mCherry* for cells were from colonies grown on supplemented Lee’s Glc agar or GlcNAc agar under conditions noted on the left. Scale bars,10 μm.

To test whether Nie et al. (18) had in fact analyzed gray rather than opaque cells, we repeated their core experiments, employing the conditions and media reported in their publication, employing the double mutants **a/a** P37005 *efg1*Δ/Δ *wor1*Δ/Δ and −/α SC5314 *efg1*Δ/Δ *wor1*Δ/Δ, which were transformed with *GFP* under the control of the gray-specific *HSP31* promoter and m*Cherry* under the control of the opaque-specific *OP4* promoter. Cells of those two strains grown in suspension in yeast extract-peptone-glucose (YPD) medium at 25°C in air at pH 6.8 (18) expressed *GFP* in log phase and at a higher level in stationary phase but not m*Cherry* in either phase (Fig. 3A), indicating that the cells studied by Nie et al. (18) were in the gray, not opaque, phase (Fig. 3A and B). Since Nie et al. (18) found that the opaque-like cells they analyzed formed in synthetic defined medium (SD) suspension cultures at pH 6.8 but not pH 4.5, we performed the experiment at both pHs. Only the pH 6.8 cultures expressed *GFP* but not *mCherry* (Fig. 3B), again indicating that the cells they analyzed expressed the gray phenotype. At pH 4.5, the double mutants formed white cells expressing neither *GFP* nor *mCherry* (Fig. 3B). It should be noted that the cell size and morphologies were those of the gray “intermediate” phenotype (15–17, 19) not the opaque phenotype (Fig. 3A and B). The white, gray, and opaque phenotypes formed on Glc agar, pH 6.8, at 25°C in air, on GlcNAc agar, pH 6.8, at 25°C in air, and at 37°C in 5% CO_2_, respectively, are presented in Fig. 3C for comparison.

### Stability of *efg1*Δ/Δ *wor1*Δ/Δ opaque cells.

**FIG 4:**
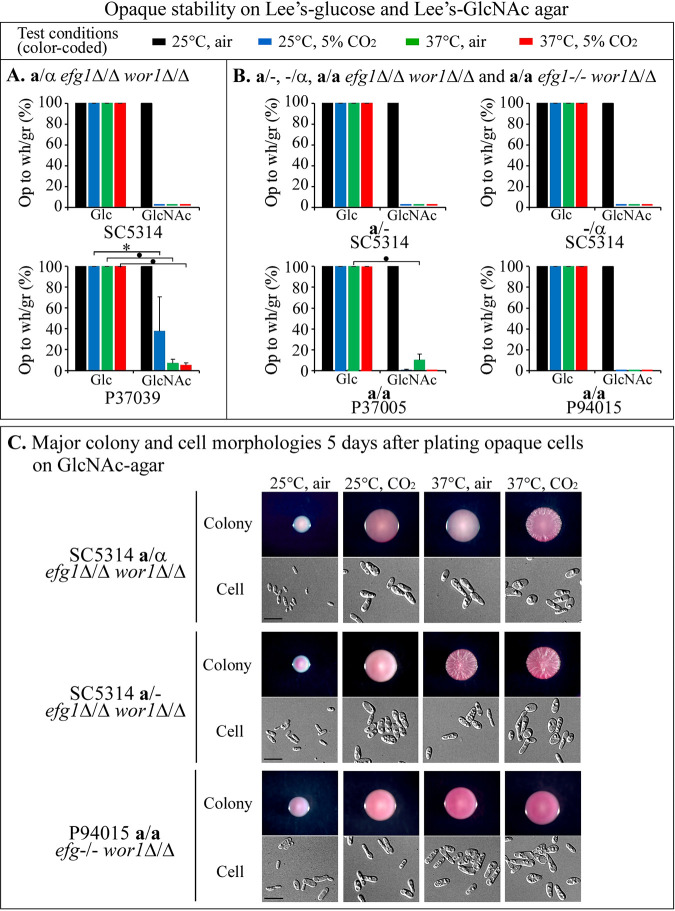
Opaque cell stability of *efg1*Δ/Δ *wor1*Δ/Δ double mutants. The stability of opaque cells was assessed by measuring the frequency of switching from opaque to white or gray when opaque cells were plated on Glc agar and GlcNAc agar under the four tested sets of conditions (top). (A) Stability of opaque cells formed by SC5314 and P37039 **a**/α *efg1*Δ/Δ *wor1*Δ/Δ double mutants. •, *P* value < 1 × 10^−6^; *, *P* value = 0.004. (B) Stability of opaque cells formed by SC5314 **a**/−, SC5314 −/α, and P37005 **a/a** *efg1*Δ/Δ *wor1*Δ/Δ double mutants and the P94015 **a/a** *efg1^−/−^ wor1*Δ/Δ double mutant. On average, >500 colonies were assessed in each of three experiments. Error bars represent the standard deviations of the means from the three experiments. Colonies with sectors containing white and gray cells were counted as nonopaque. (C) Colony and cell morphologies formed by opaque cells of double mutants *efg1*Δ/Δ *wor1*Δ/Δ or *efg1^−/−^ wor1*Δ/Δ plated on GlcNAc agar under the four tested sets of environmental conditions. Representative images were captured 5 days after plating of opaque cells. Scale bars, 10 μm.

We previously demonstrated that opaque cells of **a**/α *efg1*Δ/Δ *WOR1^+/+^* mutants were stable on Glc agar at 25°C in air or 5% CO_2_ but unstable at 37°C in air or 5% CO_2_ (19). On GlcNAc agar, opaque cells were relatively stable under all four sets of conditions (19). We therefore similarly tested opaque cell stability of all six **a**/α, **a**/−, −/α, and **a/a** *efg1*Δ/Δ *wor1*Δ/Δ or *efg1^−/−^ wor1*Δ/Δ double mutants; opaque cells obtained from opaque colonies formed on GlcNAc agar at 37°C in 5% CO_2_. On Glc agar, opaque cells of the **a**/α, **a**/−, −/α, and **a/a** double mutants were unstable under all four sets of conditions (Fig. 4A and B). On GlcNAc agar, none of the six double mutants were stable at 25°C in air (Fig. 4A and B), all switching to the gray phenotype (Fig. 4C). However, on GlcNAc agar at 25°C in 5% CO_2_ and at 37°C in both air and 5% CO_2_, all of the **a/a**, **a**/−, −/α, and **a/a** double mutants were relatively stable (Fig. 4A to C). These results demonstrate that opaque cells of the double mutants were less stable than the opaque cells of the *efg1*Δ/Δ *WOR1^+/+^* single mutants (19).

### Mating competence.

**FIG 5:**
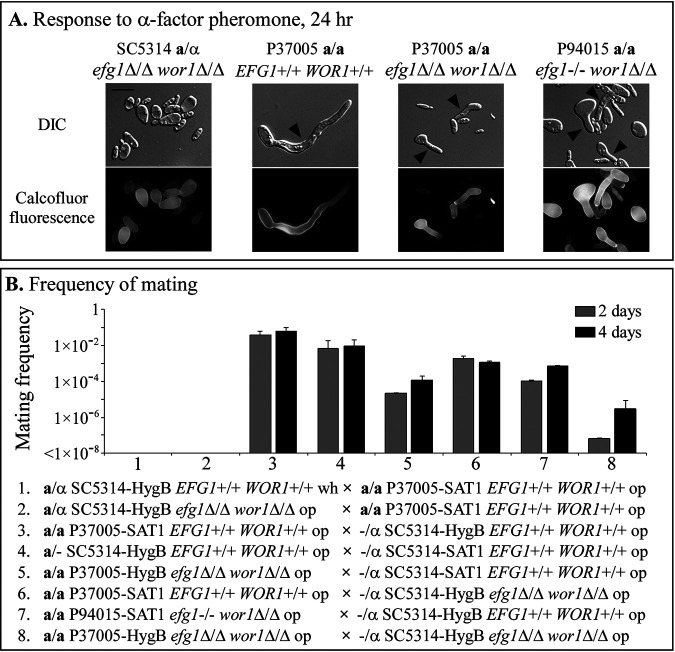
Opaque cells **a**/−, −/α, and **a**/**a** double mutants are mating competent. (A) Conjugation tube formation in response to α-pheromone in supplemented Lee’s glucose medium at 25°C in air after 24 h. Cells were stained with calcofluor to assess the formation of conjugation tubes by the absence of a septum. (B) Mating frequencies of eight crosses on Glc agar, incubated for 2 and 4 days at 25°C in air. In each cross, one strain was resistant to hygromycin B and the other to nourseothricin. Scale bar, 10 μm. HygB, resistance gene to hygromycin B; SAT1, resistance gene to nourseothricin; wh, white; op, opaque.

The **a**1-α2 corepressor represses both white-opaque switching and mating in **a**/α cells (3). In **a**/**a** and α/α cells, one of the two proteins of the corepressor is missing, and cells can therefore mate, but with one caveat. They must first switch from white to the opaque phenotype to be mating competent (3, 28). Although select **a**/α clinical strains harboring mutations in genes that repress switching can be induced to form opaque cells, these **a**/α opaque cells do not respond to pheromone by forming conjugation tubes, nor do they mate, presumably due to the presence of the **a**1-α2 repressor (14). Opaque cells formed by SC5314 **a**/α *efg1*Δ/Δ *wor1*Δ/Δ double mutants treated with α-pheromone did not form conjugation tubes (Fig. 5A). Opaque cells formed by wild-type P37005 **a/a** *EFG1^+/+^ WOR1^+/+^* treated with α-pheromone, however, formed long conjugation tubes by 24 h (Fig. 5A). Opaque cells of two representative double mutants, P37005 **a/a** *efg1*Δ/Δ *wor1*Δ/Δ and P94015 **a/a** *efg1^−/−^ wor1*Δ/Δ, formed conjugation tubes in response to α-pheromone, although the tubes were noticeably shorter than those of wild-type P37005 **a/a** *EFG1^+/+^ WOR1^+/+^* opaque cells (Fig. 5A).

To test for mating, we employed a strategy in which mating partners were individually resistant to hygromycin B and nourseothricin, as described in a previous study (30). Opaque cells of **a**/α SC5314 *efg1*Δ/Δ *wor1*Δ/Δ did not mate with opaque cells of **a/a** P37005 *EFG1^+/+^ WOR1^+/+^* (Fig. 5B). Like opaque cells of *MTL*-hemizygous and *MTL*-homozygous *EFG1^+/+^ WOR1^+/+^* wild-type strains, opaque cells of double mutants did mate with wild-type strains and *efg1*Δ/Δ *wor1*Δ/Δ double mutants, although at reduced rates (Fig. 5B).

### Gastrointestinal colonization.

**FIG 6:**
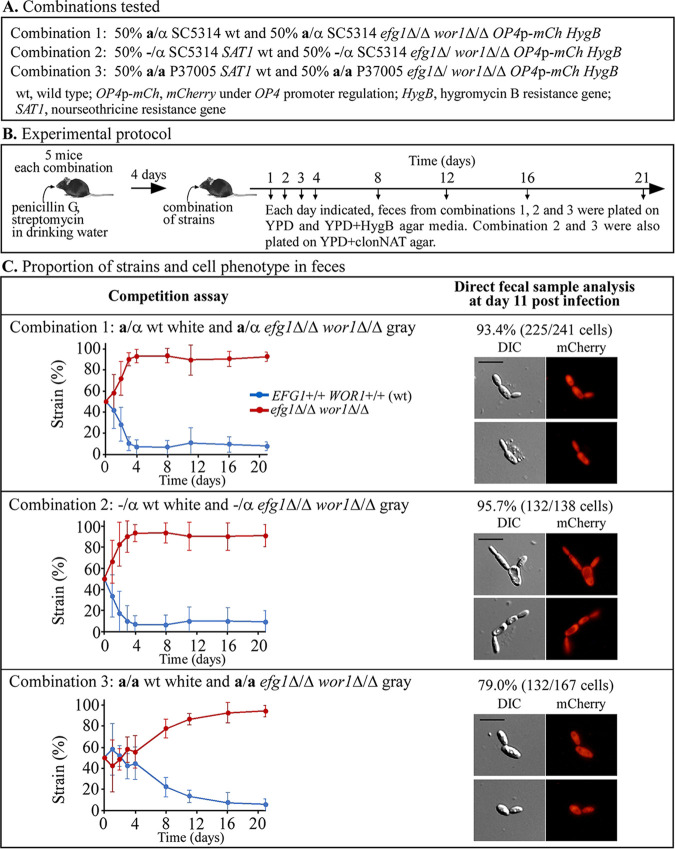
Competition experiments between wild-type (*EFG1^+/+^ WOR1^+/+^*) **a**/α, −/α, and **a**/**a** strains and their *efg1*Δ/Δ *wor1*Δ/Δ double mutants revealed that the double mutants outcompeted wild-type strains and that the resulting colonizing double mutants expressed the opaque phenotype at the site of colonization in the mouse gastrointestinal model. (A) The combinations tested for competition. (B) Experimental protocol. (C) Proportions of strains and cellular phenotypes in feces, the former assessed by plating on selection medium and the latter by microscopic analysis. The proportion of cells exhibiting the opaque phenotype and expressing *mCherry* regulated by the opaque-specific *OP4* promoter is provided as percent cells, with the number assessed in parentheses for 11-day fecal sample. Scale bars, 10 μm. HygB, hygromycin B; clonNAT, nourseothricin.

It was previously demonstrated that *efg1*Δ/Δ deletion derivatives and *efg1^−/−^* clinical strains, regardless of the original cell phenotype (i.e., white, gray, or opaque) at the time of ingestion outcompeted **a/**α *EFG1^+/+^* cells in the mouse model for gastrointestinal (GI) colonization (15, 17, 31–34). It was also demonstrated that colonizing **a**/α *efg1*Δ/Δ mutants expressed the opaque phenotype at the site of colonization (15). Here, we performed similar competition experiments between **a**/α, −/α, and **a/a** *EFG1^+/+^ WOR1^+/+^* cells (wild type) and their *efg1*Δ/Δ *wor1*Δ/Δ double deletion derivatives (Fig. 6A) to assess whether the additional deletion of *WOR1* affected the competitive advantage afforded by deletion of *EFG1* and the colonizing opaque phenotype. The genotype of colonizing cells as well as the cellular phenotype were also assessed, the former by the selection protocol and the latter by light and fluorescence microscopic observation (15). In each combination of strains, the double mutant harbored the fluorescent reporter gene *mCherry* regulated by the opaque-specific *OP4* promoter (Fig. 6A and Table S1). This allowed discrimination microscopically of opaque cells in fecal samples (15). The competitive pairs are presented in Fig. 6A and the experimental protocol in Fig. 6B. In competition experiments for combination 1 and 2, the double mutants increased to >90% of the colonizing population by day three postingestion (Fig. 6C). Cells of the P37005 **a/a** *efg1*Δ/Δ *wor1*Δ/Δ double mutant began outcompeting wild-type cells only after 3 days but reached 80% after day 16 (Fig. 6C). The proportion of opaque cells that were fluorescent (i.e., expressed *mCherry* due to activation of the opaque-specific *OP4* promoter) in the fecal samples of mice after 11 days for the three combinations were 93%, 96%, and 79%, respectively (Fig. 6C). These results indicate that the additional deletion of *WOR1* did not affect the advantage provided by deletion of *EFG1* or the dominant opaque phenotype at the site of colonization.

## DISCUSSION

### Double mutants of *EFG1* and *WOR1* form opaque cells.

Although the complex white-opaque phase transition is affected by a number of environmental conditions (29, 35–37) and appears to involve an ever-expanding list of signal transduction molecules (35, 36, 38), *trans*-acting factors (12, 39, 40), and chromatin modulators (11, 41, 42), an underlying general perception has emerged that the yin-yang expression of the TFs *EFG1* and *WOR1* plays a central regulatory role. Therefore, one might expect that deletion of both *EFG1* and *WOR1*, in a double mutant, would result in loss of the capacity of white cells, or gray cells, to switch to the opaque phenotype when transferred to the most optimum opaque-inducing conditions (GlcNAc as sugar source, 37°C, and 5% CO_2_). Indeed, reports by three different research groups, Hnisz et al. (11) in 2009, Tao et al. (16) in 2014, and Liang et al. (17) in 2019, indicated that the double mutants were locked in the white, or gray, phenotype. However, Nie et al. (18) reported in 2010 that an *efg1*Δ/Δ *wor1*Δ/Δ double deletion mutant formed opaque-like cells at pH 6.8, in air, at 22°C in studies in which glucose was employed as the carbon source. The double mutant, though elongate, was of similar size to that of white cells, consistent with the gray phenotype (16, 19); although it expressed the opaque-specific gene *OP4*, it did so at a dramatically reduced level (18), which was consistent with expression by gray cells (16). Moreover, Nie et al. (18) employed medium containing 2% glucose, which is inhibitory to opaque cell formation by *efg1^−/−^* or *efg1*Δ/Δ mutants (15, 19) at 25°C in air but which did support gray cell formation. The presence of complex cell wall pimples, present exclusively in the walls of opaque cells (26) but not gray cells (15, 19), was not assessed by Nie et al. (18). Therefore, it was unclear if *efg1*Δ/Δ *wor1*Δ/Δ double mutants were capable of forming bona fide opaque cells. For that reason, we repeated the experimental protocols of Nie et al. (18) and found that they indeed resulted in the formation of gray, not opaque, cells. Therefore, the question remained unanswered as to whether *efg1*Δ/Δ *wor1*Δ/Δ double mutants could in fact form bona fide opaque cells.

We demonstrate here that two *MTL*-heterozygous, two *MTL*-hemizygous, and two *MTL*-homozygous double mutants can be induced to form a majority of opaque cells on GlcNAc agar at 37°C in 5% CO_2_. There were distinct differences in the patterns of environmental conditions that induced the opaque phenotype between the double mutants of the two mating-type configurations. However, all six tested double mutants underwent mass conversion under the set of conditions that was most representative of conditions in the lower GI tract, namely, agar containing GlcNAc rather than glucose as the sugar source, a temperature of 37°C rather than 22 or 25°C, and a CO_2_ level of 5% rather than 0.04% CO_2_ (air). Opaque cells formed by all of the tested double mutants proved to be stable when retransferred to GlcNAc agar at 25°C in 5% CO_2_, at 37°C in air, and at 37°C in 5% CO_2_ but were unstable on glucose under all four sets of conditions. The patterns of stability under the different sets of environmental conditions on Glc agar and GlcNAc agar did differ from those previously reported for **a**/α *efg1*Δ/Δ *WOR1^+/+^* single mutants (19). Deletion of *WOR1* in addition to *EFG1* resulted in instability on Glc agar under three sets of environmental conditions. These results indicate that *WOR1* plays a conditional role in the stability of the opaque phenotype.

### Models for the regulation of switching.

A variety of genes (43) encoding signaling proteins (35, 36, 38), *trans*-acting factors (12, 39, 40), and chromatin modulation factors (11, 41, 42) play roles in regulating switching from white to opaque. Central to many of the proposed regulatory models are the two TFs. Central to many models for the regulation of white-opaque switching are the roles of *EFG1* and *WOR1* (9, 10, 12, 44). Mutants of *EFG1* are blocked in the opaque phase (5, 15, 19), and mutants of *WOR1* are blocked in the white phase (6–8). The original studies that identified *WOR1* (6–8) and many subsequent studies suggest that increased *WOR1* expression is essential for the transition to opaque.

**FIG 7:**
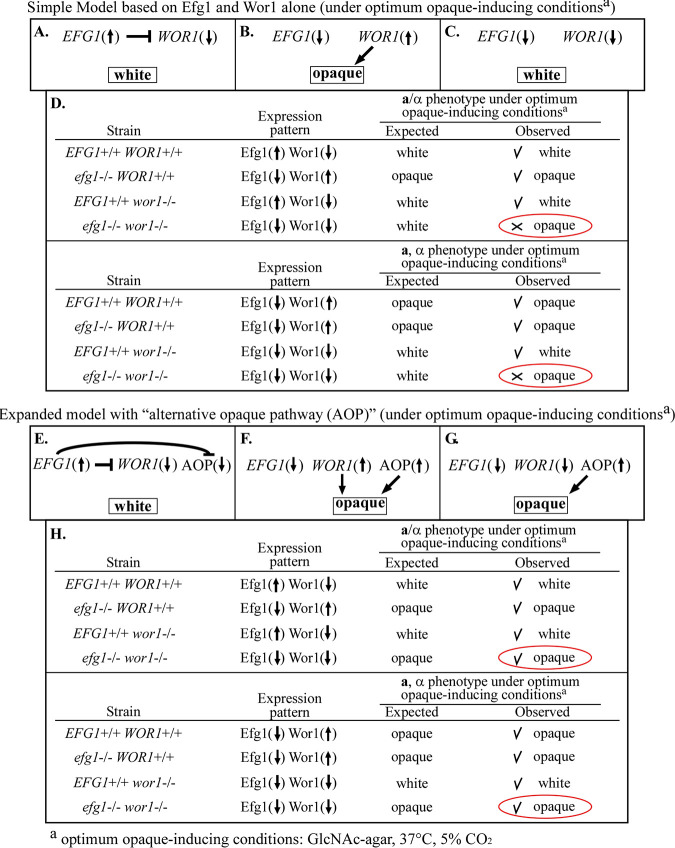
Mutant results are consistent with a model for the regulation of white-opaque switching that includes an “alternative opaque pathway” (AOP) repressed by *EFG1*. (A to C) A simple model based on the activities of *EFG1* and *WOR1* alone for cells incubated under the most optimum opaque-inducing conditions (GlcNAc agar, 37°C, 5% CO_2_). (D) The expected phenotype based on the simple model and observed phenotypes of wild-type and mutant strains. (E to G) An expanded model based on the activities of *EFG1*, *WOR1*, and AOP. (H) The expected and observed phenotypes based on the expanded model for cells under the most optimum opaque-inducing conditions of the wild-type and mutant strains. Arrows in parentheses pointing up denote upregulation and arrows in parentheses pointing down denote downregulation. Checkmarks next to observed phenotypes indicate that the “observed” is consistent with “expected,” based on respective models. Crosses next to one observed phenotype (*efg1^−/−^ wor1^−/−^*) for the simple model indicate that the observed phenotype is inconsistent with the expected one.

Under *in vitro* conditions that are most consistent with the biological conditions of the lower GI tract (GlcNAc agar at 37°C in 5% CO_2_), the simplest model contains only two components, *EFG1* and *WOR1* (Fig. 7A to C). When *EFG1* is upregulated, it represses *WOR1* expression (Fig. 7A). Since *WOR1* expression activates the opaque phenotype, repression or deletion of *WOR1* results in constitutive expression of the white phenotype (Fig. 7A). When *EFG1* is downregulated, *WOR1* is derepressed, activating genes directly involved in the structural and physiological changes associated with expression of the opaque phenotype (Fig. 7B). In the absence of both *EFG1* and *WOR1* expression, the model predicts that the white phenotype will continue to be expressed under the defined set of physiological conditions, due to the absence of *WOR1* activation (Fig. 7C). In Fig. 7D, we have summarized the “expected” phenotypes based upon this simple model (Fig. 7A to C) and “observed” phenotypes obtained in our plating experiments (Table 1) for **a**/α strains and for **a** and α *EFG1^+/+^ WOR1^+/+^* strains, *efg1^−/−^ WOR1^+/+^* mutants, *EFG1^+/+^ wor1^−/−^* mutants, and *efg1^−/−^ wor1^−/−^* mutants. The observed phenotype was the same as the expected phenotype for wild-type *EFG1^+/+^ WOR1^+/+^* strains, the *efg1^−/−^ WOR1^+/+^* single mutants, and the *EFG1^+/+^ wor1^−/−^* single mutants but differed for the *efg1^−/−^ wor1^−/−^* double mutants (Fig. 7D). The expected phenotype for the double mutant was white given the absence of the activator Wor1 (Fig. 7C and D), but the observed phenotype was opaque (Fig. 7D). This inconsistency between expected and observed phenotypes of the double mutants is accommodated by expanding the model to include an alternative opaque pathway that activates the opaque phenotype (AOP) (Fig. 7E to G). In the expanded model, when *EFG1* is upregulated, it represses *WOR1* and the alternative opaque activator pathway (AOP), thus blocking the activation of the white-to-opaque transition (Fig. 7E). When *EFG1* is downregulated, *WOR1* and AOP are derepressed, thus activating the transition from white to opaque (Fig. 7F). In the absence of both *EFG1* and *WOR1*, AOP is derepressed and it alone activates the transition to opaque (Fig. 7G). In Fig. 7H, we have summarized the expected and observed phenotypes based on the alternative model described in Fig. 7E to G for wild-type and mutant strains under optimum opaque-inducing conditions. In this expanded model, the observed was the same as expected for all wild-type and mutant strains, including, most notably, the double mutant (Fig. 7H).

Although the expanded model (Fig. 7H) accounts for the phenotypes of wild-type and all mutant strains, including the double mutants, under optimum opaque-inducing conditions, it is very likely an oversimplification, based upon a number of observations. First, it is clear that *EFG1* is not the only repressor of white-to-opaque switching. In addition to deletion of *EFG1*, Xie et al. (14) found that deletion of the TF genes *RFG1* and *BRG1* and Park et al. (19) found that deletion of the TF gene *SFL2* derepressed the white-to-opaque switch in **a**/α *EFG1^+/+^ WOR1^+/+^* cells. Moreover, in a recent analysis of 27 **a**/α clinical isolates, close to one-half (13 isolates) switched to opaque, but only approximately one-half of the latter isolates were *efg1^−/−^* mutants (15). The other five **a**/α switchers were *EFG1^+/+^* or *EFG1^+/−^* and thus presumably harbored a mutation in one or more genes other than *EFG1* that function as repressors of white-to-opaque switching (15). Moreover, of the 13 clinical isolates that switched (eight *efg1^−/−^*, one *EFG1^+/−^*, and four *EFG1^+/+^* clinical isolates), only seven were complemented with a functional copy of *EFG1*, and all seven were *efg1^−/−^* (15), supporting the conclusion that there are repressors of switching other than Efg1. Presumably, these genes may also be repressors of *WOR1* and AOP. In addition to the complexity of repressor genes, the role of the mating-type locus has not been fully explored nor has the role of other genes deemed essential for switching (39, 41). Finally, the model does not take into consideration the role of chromatin modifiers (11, 42, 45, 46).

One observation we have made, however, is clear. In the simultaneous absence of Efg1 and Wor1 (double mutants *efg1^−/−^ wor1^−/−^*), *MTL*-heterozygous, *MTL*-hemizygous, and *MTL*-homozygous white or gray cell populations can be induced to switch *en masse* to the opaque phenotype. If two different regulatory pathways function upstream of the many genes involved in the generation of the complex opaque phenotype, they may do so either by individually targeting each of the promoters of those genes or by targeting a single regulator, which in turn activates and represses a common set of genes basic to the phenotypic transition. The latter scenario seems far more efficient and less vulnerable to mutations that would affect the integrity of the opaque phenotype, especially in relation to its role in mating. The final genes targeted by the regulatory network downstream of *WOR1* and the AOP are most likely involved in such processes as the generation of the complex, opaque-specific cell wall pimple (25, 26), the release of opaque-specific exosomes (26), the formation of an enlarged vesicle-filled vacuole (25, 26), a change in white blood cell phagocytosis (47), loss of the release of a leukocyte chemoattractant (48), a switch from a gene expression profile consistent with fermentative metabolism (white) to a profile consistent with oxidative metabolism (opaque) (49), alternative roles in a pheromone-based paracrine system for generating a sexual biofilm (50), and many more opaque-specific characteristics. The role of the upstream *WOR1* and AOP networks may be to activate the white-opaque transition in response to different environmental cues (15, 19, 51). Our results, summarized in Table 1, add weight to this suggestion. If the *WOR1* network and the AOP network were purely redundant, then the *efg1^−/−^ WOR1^+/+^* and *efg1^−/−^ wor1^−/−^* mutants would have the same pattern of phenotypic responses to the four sets of environmental conditions on GlcNAc agar. This was not the case for the *MTL*-hemizygous and *MTL*-homozygous strains at 25°C in air and the **a**/α strains at 25°C in 5% CO_2_ and 37°C in air (Table 1). These results suggest that the *WOR1* network and the unidentified AOP network may be responding to environmental conditions differently and that, under select conditions, either *WOR1* or AOP activates the opaque phenotype (Table 1). These results in turn suggest that the alternative activation pathways may respond to very different physiological conditions within the host. The next challenges in elucidating the mechanisms regulating the white-opaque transition therefore will be to identify the “alternative opaque pathway” and test for the existence of the hypothesized common downstream regulator, which activates those genes that are directly involved in the structural and physiological changes associated with the generation of the opaque phenotype.

## MATERIALS AND METHODS

### Strains and media.

The C. albicans strains used in this study are described in Table S1 in the supplemental material. The strains were maintained at room temperature on agar containing YPD medium (1% yeast extract, 2% peptone, 2% glucose). Escherichia coli strain XL1-Blue (Agilent Technologies, TX, USA), used to maintain plasmids, was grown in LB medium (1% tryptone, 0.5% yeast extract, 1% NaCl) plus 100 μg/ml ampicillin.

### Plasmids and construction of C. albicans strains.

Plasmids pEFG1SM and pWOR1SM (19) were used to delete *EFG1* and *WOR1*, respectively. The plasmid pEFG1C (19), which contains a wild-type copy of *EFG1* obtained from strain SC5314 (sc*EFG1*), was used to complement the clinical isolate P94015 **a/a** *efg1^−/−^* and its *wor1*Δ/Δ derivative to generate the derivatives P94015 **a/a** *EFG1^+/−^ WOR1^+/+^* and P94015 **a/a** *EFG1^+/−^ wor1*Δ/Δ. Plasmids pmCherry-HygB (19) and pGFP-SAT (52) were used to create strains harboring the *mCherry* and *GFP* genes, respectively, under the regulation of the opaque-specific *OP4* promoter. Plasmid pHSP31-GFPS was generated by swapping 5′ and 3′ *OP4* fragments in the plasmid pGFP-SAT for 5′ and 3′ *HSP31* fragments, which were amplified by PCR from genomic DNA of SC5314 strain with primer pairs HSP31-5F/-5R and HSP31-3F/-3R (see Table S2), respectively. The *CaSAT1* gene was used to confer resistance to nourseothricin (clonNAT), and the *CaHygB* gene was used to confer resistance to hygromycin B (HygB). For selection of cells transformed with the gene deletion and insertion cassettes of the plasmids noted above and described in previous studies (15, 19), YPD agar containing 200 μg/ml of nourseothricin or 1 mg/ml of hygromycin B, respectively, was used. Integration into proper loci was verified by PCR by using the relevant primers listed in Table S2.

10.1128/mSphere.00918-20.2 TABLE S2 Primers used in this study. Download Table S2, PDF file, 0.3 MB. Copyright © 2020 Park et al. 2020 Park et al. This content is distributed under the terms of the Creative Commons Attribution 4.0 International license.

### Switching assay.

Switching was tested according to procedures recently described in detail (15, 19). In brief, cells were plated on agar containing the ingredients of Lee’s medium at pH 6.8 (23, 24) supplemented with either 1.25% glucose (“Glc agar”) or 2% *N*-acetylglucosamine (“GlcNAc agar”) as the sugar source. The agar was supplemented with 5 μg/liter of phloxine B, which stains opaque colonies light pink to red (25). Eight sets of environmental conditions were tested, which included all combinatorial permutations of sugar source (1.25% glucose versus 2% GlcNAc), temperature (25°C versus 37°C), and atmosphere (air [0.04% CO_2_] versus 5% CO_2_). Cells from YPD suspension cultures, grown overnight at 25°C, were plated on Glc agar or GlcNAc agar plates at approximately 200 cells per 10-cm petri dish. Colony phenotypes were typically analyzed after 5 days of incubation. Homogeneous opaque colonies containing exclusively opaque cells and white colonies with major opaque sectors containing almost exclusively opaque cells were counted as “opaque.” Representative colonies were analyzed for cellular phenotypes as described previously (19). Switching experiments were repeated three times; each repeat included >500 colonies. The means ± standard deviations were computed for each set of three repeats. To assess opaque cell stability, cells from homogeneous opaque colonies formed on GlcNAc agar at 37°C in 5% CO_2_ were replated on Glc or GlcNAc agar plates and analyzed for colony phenotype under the four sets of environmental conditions (25°C in air, 25°C in 5% CO_2_, 37°C in air, and 37°C in 5% CO_2_) as described above.

### Immunostaining of the opaque-specific pimple.

The procedure was described previously in detail (15, 19). In brief, cells were heat killed in a 65°C water bath for 1 h, pelleted, and resuspended in phosphate buffer solution (PBS) supplemented with 10% normal goat serum to block nonspecific binding. A 1:50 dilution of the rabbit-derived polyclonal anti-pimple antiserum (25, 26) was preabsorbed five times with heat-killed *MTL*-homozygous white cells to remove antibodies to surface antigens common to white and opaque cells. Cells were then washed with PBS and treated with Alexa Fluor 488-tagged goat anti-rabbit secondary antibody (Jackson ImmunoResearch, West Grove, PA). Differential interference contrast (DIC) and fluorescence images were captured using a Canon Rebel T3i digital camera attached to a Nikon-PE2000 inverted microscope through a 60× plan water immersion lens objective.

### Mating assay.

To assess the mating capacity of opaque cells, strains were used that constitutively expressed genes that conferred resistance to either hygromycin B or nourseothricin (Table S1; Fig. 5C). To measure mating, cell mixtures were obtained from 5 to 10 homogeneous white or opaque colonies per partner strain. For crosses, white and opaque colonies of the parental wild-type strains were grown for 5 days on Glc agar at 25°C in air, whereas opaque colonies of double mutants were grown for 5 days on GlcNAc agar at 37°C in 5% CO_2_. Ten microliters containing 3 × 10^6^ cells of a mating mixture was spotted on a nitrocellulose membrane placed on Glc agar and incubated for 2 and 4 days at 25°C in air. Cells in the mating patch were then resuspended in 1 ml of water, and 100-μl aliquots of undiluted and three 10-fold serial dilutions were plated on YPD selection agar, which contained 200 μg/ml of nourseothricin and 1 mg/ml of hygromycin B. To measure total CFU in the recovered mating suspensions, 100-μl aliquots of 10^−4^ and 10^−5^ dilutions were plated on YPD agar without selection reagents. For both selective and nonselective conditions, CFU were measured after 3 days of incubation at 30°C, and mating frequencies were plotted.

### Repeat of Nie et al. (18) culture conditions.

To distinguish between opaque and gray cells under the conditions of Nie et al. (18), we generated SC5314 −/α *efg1*Δ/Δ *wor1*Δ/Δ and P37005 **a**/**a** *efg1*Δ/Δ *wor1*Δ/Δ double deletion mutants that harbored an *mCherry* gene regulated by the opaque-specific *OP4* promoter (53) and a *GFP* gene regulated by the gray-specific *HSP31* promoter (16). The mutants were grown in liquid YPD medium at pH 6.8 and SD (synthetic defined medium; 6.7 g/liter Difco yeast nitrogen base, 2% glucose) at pH 6.8 or pH 4.5 at 25°C in air. Cells from the suspension cultures grown to log phase (optical density at 600 nm [OD_600_] of 1 to 2.5) and stationary phase (OD_600_ of >8) were imaged to determine cell morphologies and expression of *GFP* and *mCherry*.

### Pheromone response assay.

Opaque cells of the wild-type strains were obtained from colonies grown for 5 days on Glc agar at 25°C in air. Opaque cells of mutant strains were obtained from colonies grown for 5 days on GlcNAc agar at 37°C in 5% CO_2_. Ten micrograms of α-factor (chemically synthesized 13mer) was added to a 1-ml cell suspension containing 2 × 10^6^ cells in supplemented Lee’s medium and incubated in the wells of a 12-well tissue culture plate for 24 h at 25°C in air. Opaque cells incubated under the same condition, but without α-factor, served as controls. At designated time points, cells from the test cultures were fixed in 2% paraformaldehyde and stained with calcofluor (Fluorescent Brightener 28; Sigma). DIC and fluorescence images were captured using a Canon Rebel T3i digital camera attached to a Nikon-PE2000 inverted microscope through a 60× plan water immersion lens objective.

### Imaging colonies and cells.

Colonies grown on agar plates were imaged through a stereomicroscope equipped with a Nikon E990 digital camera. DIC and fluorescence microscopic images of cells of colonies and cells expressing *mCherry* were obtained with a Canon Rebel T3i digital camera attached to a Nikon-PE2000 inverted microscope through a 60× plan water immersion lens objective.

### Mouse gastrointestinal tract colonization.

The methods used were previously described in detail (15). All procedures complied with regulatory guidelines defined by the Iowa University IACUC committee. C57BL/6J (Jackson Laboratories) female mice 6 to 7 weeks old were treated with 1 mg/ml of penicillin G and 2 mg/ml of streptomycin in their drinking water for 4 days (Fig. 6B). Mice were then orally inoculated with 3 × 10^7^ cells in 0.2 ml of PBS. To assess levels of colonization, competition, and phenotypic switching, fresh fecal samples were collected postingestion at time intervals up to 21 days. Samples were homogenized in 0.2 ml of water per fecal pellet, and serial dilutions were plated on YPD, YPD plus nourseothricin (YPD+clonNAT), and YPD plus hygromycin B (YPD+HygB) agar plates containing 50 μg/ml of chloramphenicol. Total CFU on nonselection agar and CFU on selection agar plates were scored after 3 days of incubation at 25°C in air. The means ± standard deviations were computed for the data from five mice. To assess cellular phenotypes in fecal samples, fecal pellets were diluted 1:10 with distilled H_2_O and examined by differential interference contrast (DIC) and fluorescence microscopy, the latter for expression of *mCherry* under regulation of the opaque-specific gene *OP4* promoter.
