# Supplementary material for: Candida albicans Double Mutants Lacking both EFG1 and WOR1 Can Still Switch to Opaque
Source: mSphere. 2020 Sep 23;5(5):e00918-20. doi: 10.1128/mSphere.00918-20 (PMC7568642; doi:10.1128/mSphere.00918-20)
Supplement: TABLE S1 [file mSphere.00918-20-st001.pdf]

TABLE S1 Strains used in this study.

Strain	Parental strain	*MTL*	Genotype	Reference
SC5314	-	**a**/α	Wild type	(1)
SC5314 *efg1*Δ/Δ	SC5314	**a**/α	*efg1*::*FRT*/*efg1*::*FRT*	(2)
SC5314mChH *efg1*Δ/Δ	SC5314 *efg1*Δ/Δ	**a**/α	*efg1*::*FRT*/*efg1*::*FRT*, *OP4*/*op4*::*OP4*p-*mCherry-CaHygB*	(2)
SC5314 *wor1*Δ/Δ	SC5314	**a**/α	*wor1*::*FRT*/*wor1*::*FRT*	This study
SC5314 *efg1*Δ/Δ *wor1*Δ/Δ	SC5314 *efg1*Δ/Δ	**a**/α	*efg1*::*FRT*/*efg1*::*FRT*, *wor1*::*FRT*/*wor1*::*FRT*	This study
SC5314mChH *efg1*Δ/Δ *wor1*Δ/Δ	SC5314 *efg1*Δ/Δ *wor1*Δ/Δ	**a**/α	*efg1*::*FRT*/*efg1*::*FRT*, *wor1*::*FRT*/*wor1*::*FRT*, *OP4*/*op4*::*OP4*p-*mCherry-CaHygB*	This study
P37039	-	**a**/α	Wild type, clinical isolate	(3)
P37039 *efg1*Δ/Δ	P37039	**a**/α	*efg1*::*FRT*/*efg1*::*FRT*	(2)
P37039mChH *efg1*Δ/Δ	P37039 *efg1*Δ/Δ	**a**/α	*efg1*::*FRT*/*efg1*::*FRT*, *OP4*/*op4*::*OP4*p-*mCherry-CaHygB*	(2)
P37039 *wor1*Δ/Δ	P37039	**a**/α	*wor1*::*FRT*/*wor1*::*FRT*	This study
P37039 *efg1*Δ/Δ *wor1*Δ/Δ	P37039 *efg1*Δ/Δ	**a**/α	*efg1*::*FRT*/*efg1*::*FRT*, *wor1*::*FRT*/*wor1*::*FRT*	This study
P37039mChH *efg1*Δ/Δ *wor1*Δ/Δ	P37039 *efg1*Δ/Δ *wor1*Δ/Δ	**a**/α	*efg1*::*FRT*/*efg1*::*FRT*, *wor1*::*FRT*/*wor1*::*FRT*, *OP4*/*op4*::*OP4*p-*mCherry-CaHygB*	This study
**a**/- SC5314	SC5314	**a**/-	*MTLα1*::*FRT, MTLα2*::*FRT*	This study
**a**/- SC5314mChH	**a**/- SC5314	**a**/-	*MTLα1*::*FRT, MTLα2*::*FRT*, *OP4*/*op4*::*OP4*p-*mCherry-CaHygB*	This study
**a**/- SC5314 *efg1*Δ/Δ	SC5314 *efg1*Δ/Δ	**a**/-	*MTLα1*::*FRT, MTLα2*::*FRT*, *efg1*::*FRT*/*efg1*::*FRT*	This study
**a**/- SC5314 *wor1*Δ/Δ	SC5314 *wor1*Δ/Δ	**a**/-	*MTLα1*::*FRT, MTLα2::FRT*, *wor1::FRT*/*wor1*::*FRT*	This study
**a**/- SC5314mChH *efg1*Δ/Δ	SC5314mChH *efg1*Δ/Δ	**a**/-	*MTLα1*::*FRT, MTLα2*::*FRT*, *efg1*::*FRT*/*efg1*::*FRT*, *OP4*/*op4*::*OP4*p-*mCherry-CaHygB*	This study
**a**/- SC5314mChH *efg1*Δ/Δ *wor1*Δ/Δ	SC5314mChH *efg1*Δ/Δ *wor1*Δ/Δ	**a**/-	*MTLα1*::*FRT, MTLα2*::*FRT*, *efg1*::*FRT*/*efg1*::*FRT*, *wor1*::*FRT*/*wor1*::*FRT*, *OP4*/*op4*::*OP4*p-*mCherry-CaHygB*	This study
-/α SC5314	SC5314	-/α	*MTL**a**1**a**2*::*FRT*	This study
-/α SC5314mChH	-/α SC5314	-/α	*MTL**a**1**a**2*::*FRT*, *OP4*/*op4*::*OP4*p-*mCherry*-Ca*HygB*	This study

-/α SC5314GFPS	-/α SC5314	-/α	*MTL***a***1***a***2::FRT*, *OP4/op4::OP4*p-*GFP-CaSAT1*	This study
-/α SC5314 *efg1*Δ/Δ	SC5314 *efg1*Δ/Δ	-/α	*MTL***a***1***a***2::FRT*, *efg1::FRT/efg1::FRT*	This study
-/α SC5314 *wor1*Δ/Δ	SC5314 *wor1*Δ/Δ	-/α	*MTL***a***1***a***2::FRT*, *wor1::FRT/wor1::FRT*	This study
-/α SC5314mChH *efg1*Δ/Δ	SC5314 *efg1*Δ/Δ	-/α	*MTL***a***1***a***2::FRT*, *efg1::FRT/efg1::FRT*, *OP4/op4::OP4*p-*mCherry-CaHygB*	This study
-/α SC5314mChH *efg1*Δ/Δ *wor1*Δ/Δ	SC5314mChH *efg1*Δ/Δ *wor1*Δ/Δ	-/α	*MTL***a***1***a***2::FRT*, *efg1::FRT/efg1::FRT*, *wor1::FRT/wor1::FRT*, *OP4/op4::OP4*p-*mCherry-CaHygB*	This study
-/α SC5314 *efg1*Δ/Δ *wor1*Δ/Δ	SC5314 *efg1*Δ/Δ *wor1*Δ/Δ	-/α	*MTL***a***1***a***2::FRT*, *efg1::FRT/efg1::FRT*, *wor1::FRT/wor1::FRT*	This study
-/α SC5314GFPS *efg1*Δ/Δ *wor1*Δ/Δ	SC5314mChH *efg1*Δ/Δ *wor1*Δ/Δ	-/α	*MTL***a***1***a***2::FRT*, *efg1::FRT/efg1::FRT*, *wor1::FRT/wor1::FRT*, *OP4/op4::OP4*p-*GFP-CaSAT1*	This study
-/α SC5314mChH *efg1*Δ/Δ *wor1*Δ/Δ	SC5314mChH *efg1*Δ/Δ *wor1*Δ/Δ	-/α	*MTL***a***1***a***2::FRT*, *efg1::FRT/efg1::FRT*, *wor1::FRT/wor1::FRT*, *OP4/op4::OP4*p-*mCherry-CaHygB*	This study
-/α SC5314 OP4-mChH HSP31-GFP *efg1*Δ/Δ *wor1*Δ/Δ	-/α SC5314mChH *efg1*Δ/Δ *wor1*Δ/Δ	-/α	*MTL***a***1***a***2::FRT*, *efg1::FRT/efg1::FRT*, *wor1::FRT/wor1::FRT*, *OP4/op4::OP4*p-*mCherry-CaHygB*, *HSP31/hsp31::HSP31*p-*GFP-CaSAT1*	This study
**a**/- P37039	P37039	**a**/-	*MTLα1::FRT, MTLα2::FRT*	This study
**a**/- P37039mChH	**a**/- P37039	**a**/-	*MTLα1::FRT, MTLα2::FRT*, *OP4/op4::OP4*p-*mCherry-CaHygB*	This study
**a**/- P37039 *efg1*Δ/Δ	P37039 *efg1*Δ/Δ	**a**/-	*MTLα1::FRT, MTLα2::FRT*, *efg1::FRT/efg1::FRT*	This study
**a**/- P37039mChH *efg1*Δ/Δ	P37039mChH *efg1*Δ/Δ	**a**/-	*MTLα1::FRT, MTLα2::FRT*, *efg1::FRT/efg1::FRT*, *OP4/op4::OP4*p-*mCherry-CaHygB*	This study
**a**/- P37039mChH *efg1*Δ/Δ *wor1*Δ/Δ	P37039mChH *efg1*Δ/Δ *wor1*Δ/Δ	**a**/-	*MTLα1::FRT, MTLα2::FRT*, *efg1::FRT/efg1::FRT*, *wor1::FRT/wor1::FRT*, *OP4/op4::OP4*p-*mCherry-CaHygB*	This study
-/α P37039	P37039	-/α	*MTL***a***1***a***2::FRT*	This study
-/α P37039mChH	-/α P37039	-/α	*MTL***a***1***a***2::FRT*, *OP4/op4::OP4*p-*mCherry*-Ca*HygB*	This study
-/α P37039GFPS	-/α P37039	-/α	*MTL***a***1***a***2::FRT*, *OP4/op4::OP4*p-*GFP-CaSAT1*	This study
-/α P37039 *efg1*Δ/Δ	P37039 *efg1*Δ/Δ	-/α	*MTL***a***1***a***2::FRT*, *efg1::FRT/efg1::FRT*	This study
-/α P37039mChH *efg1*Δ/Δ	P37039 *efg1*Δ/Δ	-/α	*MTL***a***1***a***2::FRT*, *efg1::FRT/efg1::FRT*, *OP4/op4::OP4*p-*mCherry-CaHygB*	This study

-/α P37039mChH *efg1*Δ/Δ *wor1*Δ/Δ	P37039mChH *efg1*Δ/Δ *wor1*Δ/Δ	-/α	*MTL**a**1**a**2*::*FRT*, *efg1*::*FRT*/*efg1*::*FRT*, *wor1*::*FRT*/*wor1*::*FRT*, *OP4*/*op4*::*OP4*p-*mCherry-CaHygB*	This study
P37005	-	**a**/**a**	Wild type, clinical isolate	(4)
P37005 *wor1*Δ/Δ	P37005	**a**/**a**	*wor1*::*FRT*/*wor1*::*FRT*	This study
P37005mChH	P37005	**a**/**a**	*OP4*/*op4*::*OP4*p-*mCherry-CaHygB*	This study
P37005GFPS	P37005	**a**/**a**	*OP4*/*op4*::*OP4*p-*GFP-CaSAT1*	(5)
P37005 *efg1*Δ/Δ	P37005	**a**/**a**	*efg1*::*FRT*/*efg1*::*FRT*	This study
P37005mChH *efg1*Δ/Δ	P37005 *efg1*Δ/Δ	**a**/**a**	*efg1*::*FRT*/*efg1*::*FRT*, *MTLα1*::*FRT*, *OP4*/*op4*::*OP4*p-*mCherry-CaHygB*	This study
P37005 *efg1*Δ/Δ *wor1*Δ/Δ	P37005 *efg1*Δ/Δ	**a**/**a**	*efg1*::*FRT*/*efg1*::*FRT*, *wor1*::*FRT*/*wor1*::*FRT*	This study
P37005mChH *efg1*Δ/Δ *wor1*Δ/Δ	P37005 *efg1*Δ/Δ *wor1*Δ/Δ	**a**/**a**	*efg1*::*FRT*/*efg1*::*FRT*, *wor1*::*FRT*/*wor1*::*FRT*, *OP4*/*op4*::*OP4*p-*mCherry-CaHygB*	This study
P37005 OP4-mChH HSP31-GFP *efg1*Δ/Δ *wor1*Δ/Δ	P37005mChH *efg1*Δ/Δ *wor1*Δ/Δ	**a**/**a**	*efg1*::*FRT*/*efg1*::*FRT*, *wor1*::*FRT*/*wor1*::*FRT*, *OP4*/*op4*::*OP4*p- *mCherry-CaHygB*, *HSP31*/*hsp31*::*HSP31*p-*GFP-CaSAT1*	This study
P94015	-	**a**/**a**	*efg1*$^{-}$/*efg1*$^{-}$, clinical isolate	(6)
P94015sc*EFG1*	P94015	**a**/**a**	*efg1*$^{-}$/*efg1*$^{-}$::*EFG1*p-*scEFG1-CaSAT1*	This study
P94015sc*EFG1* *wor1*Δ/Δ	P94015 *wor1*Δ/Δ	**a**/**a**	*efg1*$^{-}$/*efg1*$^{-}$::*EFG1*p-*scEFG1-CaSAT1*, *wor1*::*FRT*/*wor1*::*FRT*	This study
P94015mChH	P94015	**a**/**a**	*efg1*$^{-}$/*efg1*$^{-}$, *OP4*/*op4*::*OP4*p-*mCherry-CaHygB*	This study
P94015 *wor1*Δ/Δ	P94015	**a**/**a**	*efg1*$^{-}$/*efg1*$^{-}$, *wor1*::*FRT*/*wor1*::*FRT*	This study
P94015GFPS *wor1*Δ/Δ	P94015	**a**/**a**	*efg1*$^{-}$/*efg1*$^{-}$, *wor1*::*FRT*/*wor1*::*FRT*, *OP4*/*op4*::*OP4*p-*GFP-CaSAT1*	This study

REFERENCES

1. Gillum AM, Tsay EY, Kirsch DR. Isolation of the Candida albicans gene for orotidine-5'-phosphate decarboxylase by complementation of S. cerevisiae ura3 and E. coli pyrF mutations. Molecular & general genetics : MGG. 1984;198(1):179-82.

2. Park YN, Conway K, Conway TP, Daniels KJ, Soll DR. Roles of the Transcription Factors Sfl2 and Efg1 in White-Opaque Switching in a/alpha Strains of Candida albicans. mSphere. 2019;4(2).

3. Pujol C, Messer SA, Pfaller M, Soll DR. Drug resistance is not directly affected by mating type locus zygosity in Candida albicans. Antimicrob Agents Chemother. 2003;47(4):1207-12.

4. Slutsky B, Staebell M, Anderson J, Risen L, Pfaller M, Soll DR. "White-opaque transition": a second high-frequency switching system in Candida albicans. J Bacteriol. 1987;169(1):189-97.

5. Park YN, Daniels KJ, Pujol C, Srikantha T, Soll DR. Candida albicans forms a specialized "sexual" as well as "pathogenic" biofilm. Eukaryot Cell. 2013;12(8):1120-31.

6. Wu W, Lockhart SR, Pujol C, Srikantha T, Soll DR. Heterozygosity of genes on the sex chromosome regulates Candida albicans virulence. Mol Microbiol. 2007;64(6):1587-604.
