# Supplementary material for: Candida albicans Double Mutants Lacking both EFG1 and WOR1 Can Still Switch to Opaque
Source: mSphere. 2020 Sep 23;5(5):e00918-20. doi: 10.1128/mSphere.00918-20 (PMC7568642; doi:10.1128/mSphere.00918-20)
Supplement: TABLE S2 [file mSphere.00918-20-st002.pdf]

TABLE S2 Primers used in this study.

Primer	Sequence (5´ to 3´)	Reference
CaHygB-seq5	CAGGTGCTGGTACTGTTGGTAG	This study
CaSAT1-F	GGGCACTAAGCAGACAGCTCCTTGGC	This study
EFG1-5´F	GAGAACAAAAGAAGGGCCCATTATTCATTGCAC	(1)
EFG1-seq1	AACAACCAACCAACCCTTAACCC	(2)
EFG1-seq3	CTACCAGGTCAACAAGCAGTACC	(2)
EFG1-seq6	AAAGTATATGCATGCTTACTTTCA	This study
EFG1-seg7	ATACTGTTCCCATTACCGCTAGAAC	This study
HSP31-5F	GTCAAATTGATTCTAGAAACAGTTGCGATTTC	This study
HSP31-5R	TATAGTCGACTTTTCTTTCTTTGTTAAGCCTCTTGTGGTTGC	This study
HSP31-3F	ATATCTGCAGGTAAGAGGTTGGTGAGTTCATTGGAAC	This study
HSP31-3R	TAATGAGCTCATTTTGACAGGATCGCATCT	This study
HSP31-seq	TTGGATACTCGTCGACCAATGTAC	This study
MTLa1F	TTGAAGCGTGAGAGGCAGGAG	(2)
MTLa1R	GATTAGGCTGTTTGTTCTTCTCG	(2)
MTLα2F	CATGAATTCACGTCTGGAGGCAC	(2)
MTLα2R	AAGCAGCCAACTCAGGTCAC	(2)
OP4-seq5	GACAATTTGTGAACCTCTAGCC	This study
WOR1-1	CCAAAACCCTGCCTTTCCTGTATTG	This study
WOR1-2	GAACGAGGCGTGCTTATAGTTTCTGG	This study
WOR1-3	GTACCATTGTCCATAACGCTGGAG	This study
WOR1-4	CGCAATGACAAATGGTCAAGGCGTC	This study

REFERENCES

1. Park YN, Conway K, Conway TP, Daniels KJ, Soll DR. Roles of the Transcription Factors Sfl2 and Efg1 in White-Opaque Switching in a/alpha Strains of Candida albicans. mSphere. 2019;4(2).

2. Park YN, Conway K, Pujol C, Daniels KJ, Soll DR. EFG1 Mutations, Phenotypic Switching, and Colonization by Clinical a/alpha Strains of Candida albicans. mSphere. 2020;5(1).
